# Targeted Expansion of Treg Cells to Induce Immune Tolerance after Kidney Transplantation

**DOI:** 10.1002/advs.202507943

**Published:** 2025-10-24

**Authors:** Tong Lu, Hongtao Song, Limin He, Zhihao Hu, Yu Li, Chao Xu, Shaojie Liu, Jun Jiang, Yike Zhou, Zhengxuan Li, Zeyu Li, Keying Zhang, Shuaijun Ma, Ruochen Qi, Shichao Han, Changhong Shi, Weihong Wen, Donghui Han, Weijun Qin

**Affiliations:** ^1^ Department of Urology Xijing Hospital Fourth Military Medical University Xi'an 710032 China; ^2^ Institute of Medical Research Northwestern Polytechnical University Xi'an 710072 China; ^3^ Division of Cancer Biology Laboratory Animal Center Fourth Military Medical University Xi'an Shaanxi 710032 China; ^4^ Department of Urology The 900th Hospital of Joint Logistic Support Force PLA (Fuzong Clinical Medical College of Fujian Medical University) Fuzhou 350025 China; ^5^ Department of Urology Daping Hospital Army Medical University Chongqing 400042 China; ^6^ Department of Urology The Air Force Hospital of Eastern Theater Command Nanjing 210001 China

**Keywords:** CD248/endosialin/tem‐1, immune tolerance, kidney transplantation, nanoparticles, regulatory t cells

## Abstract

Kidney transplantation remains the optimal treatment for end‐stage renal disease, yet faces persistent challenges including organ shortages and risk of infection due to systemic immunosuppression. Cell therapy is expected to replace immunosuppressive agents. However, while Treg cell therapy can mitigate immune rejection, it fails to significantly prolong graft survival because peripherally induced Treg cells exhibit transdifferentiation potential in the circulatory system. Recent advances in nanocarrier systems offer promising approaches for achieving graft‐specific immune tolerance. Through single‐cell sequencing analysis, CD248 is identified as a pivotal stromal cell marker in renal allograft rejection, modulated by HIF‐1α and IL‐1β signaling pathways. Leveraging macrophage membrane coating technology, nanoparticles co‐loaded is developed with IL‐2 and TGF‐β expressing plasmids. These nanoparticles incorporate a CD248‐targeting antibody (IgG78) and plasmids containing a kidney‐specific NPHS2 promoter, enabling dual‐targeting capability for localized gene expression. In vitro validation demonstrated efficient differentiation of CD4⁺T cells into functional Treg populations. In rat renal transplantation models, nanoparticle treatment increased Treg cells in the graft and significantly prolonged allograft survival, improved renal function, and attenuated complement deposition. The findings establish a targeted nanoparticle platform that promotes graft‐specific immune tolerance through localized Treg cell expansion, potentially reducing dependence on systemic immunosuppression.

## Graphical abstract

A dual‐targeting nanoparticle platform that selectively engages graft stromal cells via CD248 receptors while employing kidney‐ specific promoters (NPHS2) to drive localized IL‐ 2/TGF‐βexpression is developed. This approach achieves three therapeutic objectives:1) intragraft Treg polarization without systemic cytokine exposure;2) reduced dependence on nephrotoxic calcineurin inhibitors;and 3) preservation of antimicrobial immunity through partial immune system liberation—a critical advantage over conventional immunosuppression.

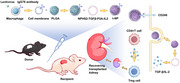

## 1 Introduction

Kidney transplantation is an important treatment for end‐stage renal disease. Compared to dialysis, kidney transplantation significantly improves patients' quality of life.^[^ ^1^, ^2^ ^]^ Despite being the gold‐standard intervention for end‐stage renal disease, persistent organ shortages constrained by ethical‐legal complexities and socioreligious considerations limits the development of renal transplantation.^[^ ^3^ ^]^ Therefore, it is important to prolong the survival time of the graft as much as possible, for renal transplant patients.

The current standard immunosuppressive protocol—combining tacrolimus, mycophenolate mofetil, and corticosteroids—presents significant clinical dilemmas. While effectively preventing acute rejection, this regimen's narrow therapeutic index exacerbates infection risks during viral outbreaks (e.g., influenza, COVID‐19) and promotes progressive renal damage through calcineurin inhibitor toxicity.^[^ ^4^ ^]^ Notably, most long‐term graft failures result from chronic rejection despite adequate immunosuppression,^[^ ^5^ ^]^ underscoring the urgent need for tolerance‐inducing therapies that minimize systemic drug exposure.

Maintaining graft homeostasis requires precise regulation of intragraft immune cell dynamics. Regulatory T cells (Tregs) play dual roles in sustaining peripheral tolerance through immune suppression and facilitating tissue repair.^[^ ^6^, ^7^ ^]^ Clinical trials have shown that Treg cell therapy significantly reduces the risk of infection in patients, but does not prolong the survival of the graft.^[^ ^8^ ^]^ While TGF‐β/IL‐2 stimulation can generate Tregs from CD4⁺ T cells in vitro, clinical translation fails due to rapid dedifferentiation upon reinfusion.^[^ ^9^ ^]^ This attrition stems from microenvironmental disparities between culture conditions and physiological compartments, highlighting the need for localized Treg expansion strategies.

Emerging nanotechnologies offer novel solutions for spatial immune modulation. Then, selecting the appropriate target is the first step to achieve the specific expansion of Treg cells in the graft.^[^ ^10^ ^]^ CD248 is a single transmembrane glycoprotein expressed on stromal cells, including glomerular cells and fibroblasts. Current research indicates that CD248 is associated with tissue fibrosis and immune cell infiltration, but studies on its role in transplants are still lacking.^[^ ^11^, ^12^, ^13^ ^]^ Our research shows that CD248 is significantly upregulated after kidney transplantation, making it an ideal target. Our research group previously developed a specific anti‐CD248 antibody, IgG78. Additionally, the biomimetic strategy of using cell membranes to coat nanoparticles is a relatively mature approach to improving bioavailability.^[^ ^14^ ^]^

In this research, we developed a dual‐targeting nanoparticle platform that selectively engages graft stromal cells via CD248 while employing a kidney‐specific promoter (NPHS2) to drive localized IL‐2/TGF‐β expression. This approach achieves three therapeutic objectives: 1) precisely targeted graft to induce Treg polarization without systemic cytokine exposure; 2) reduced dependence on nephrotoxic calcineurin inhibitors; and 3) preservation of antimicrobial immunity through partial immune system liberation—a critical advantage over conventional immunosuppression.

## 2 Results

### 2.1 CD248 is Upregulated Sustainedly in Allograft Kidney

**Figure 1:**
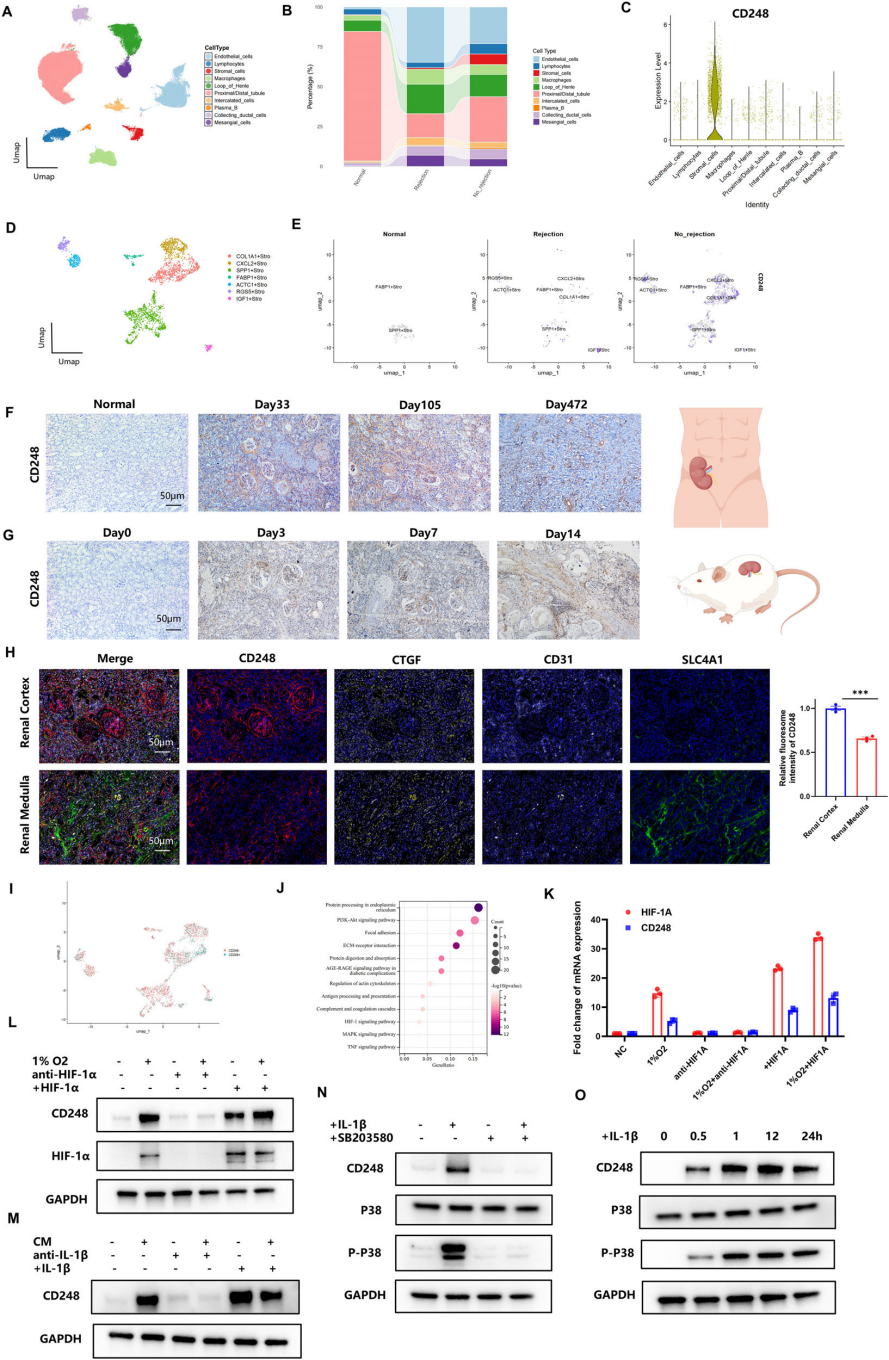
CD248 is upregulated sustainedly in allograft kidney.A) UMAP plot of the integrated data. Cells were marked using a differential color scheme. B) The cell proportion diagram of normal group, rejection group and no rejection group. C) The violin diagram shows the expression of CD248 in various types of cells. D) UMAP plot of stromal cells. E) UMAP plot for CD248 expression. F) Representative IHC staining for CD248 expression of three tissues after renal transplantation and 1 normal tissue. Scale bars: 50 µm. G) Representative IHC staining for CD248 expression of Rats after renal transplantation. Scale bars: 50 µm. H) Representative IF staining of CD248, CD31(endothelial cells), CTGF(mesangial cells) and SLC4A1(intercalated cells) to show the location of CD248. Scale bars: 50 µm. I) UMAP plot of stromal cells to show the CD248 positive cells and negative cells. J) KEEG analysis of differential genes between CD248 positive cells and negative cells. K–N) qPCR and Western blot for HIF1α‐CD248 pathway. M–O) Western blot for IL1β‐P38‐CD248 pathways.

We analyzed single‐cell sequencing data from 10 kidney transplant recipients stratified into three clinical categories: normal, rejection, and no rejection. Dimensionality reduction and clustering delineated 10 distinct cellular subpopulations: endothelial cells, lymphocytes, stromal cells, macrophages, loop of Henle cells, proximal/distal tubular cells, intercalated cells, collecting duct cells, B lymphocytes, and mesangial cells (**Figure** **1**A; Figure S1A,B, Supporting Information). Comparative analysis demonstrated greater stromal cell predominance in the non‐rejection group versus both rejection and normal cohorts (Figure 1B), implying possible graft‐protective functions. CD248 emerged as a specific stromal cell marker showing marked transcriptional upregulation in non‐rejection samples compared to other groups (Figure 1C–E; Figure S1C–E, Supporting Information).

Cell‐cell interaction mapping revealed broad immune cell engagement with parenchymal cells, excluding proximal/distal tubular elements (Figure S1F, Supporting Information). Notably, stromal cells exhibited robust paracrine signaling through collagen deposition and cytokine release (Figure S1G,H, Supporting Information), suggesting mechanistic involvement in chronic allograft adaptation processes.

To validate these observations, we procured renal allograft biopsies from three transplant recipients (Table S1, Supporting Information). Longitudinal analysis demonstrated persistent CD248 expression from 1‐month to 1‐year post‐transplant intervals (Figure 1F), a pattern corroborated in murine allograft models (Figure 1G). Spatial localization via immunofluorescence confirmed predominant CD248 expression within graft stromal cells (Figure 1H; Figure S1I, Supporting Information).

Mechanistic investigation revealed two distinct stromal subsets (CD248^+^ and CD248^−^ stromal cells) through expression‐based stratification (Figure 1I). KEGG pathway analysis identified concomitant activation of HIF‐1α and MAPK signaling cascades in CD248^+^ populations (Figure 1J). Subsequent functional validation using primary human renal stromal cells and murine counterparts demonstrated hypoxia‐induced CD248 upregulation via HIF1α‐dependent transcription, mirroring ischemia‐reperfusion pathophysiology (Figure 1K,L; Figure S2, Supporting Information).

Notably, co‐culture experiments with recipient‐derived PBMCs revealed IL1β‐mediated CD248 induction through P38‐MAPK phosphorylation (Figure 1M–O), mechanistically linking its expression to both acute and chronic rejection processes. Our multimodal approach establishes CD248 as a dynamically regulated therapeutic target throughout post‐transplant adaptation and alloimmune challenges.

### 2.2 Fabrication and Characterization of Membrane‐Coated Nanoparticles

**Figure 2:**
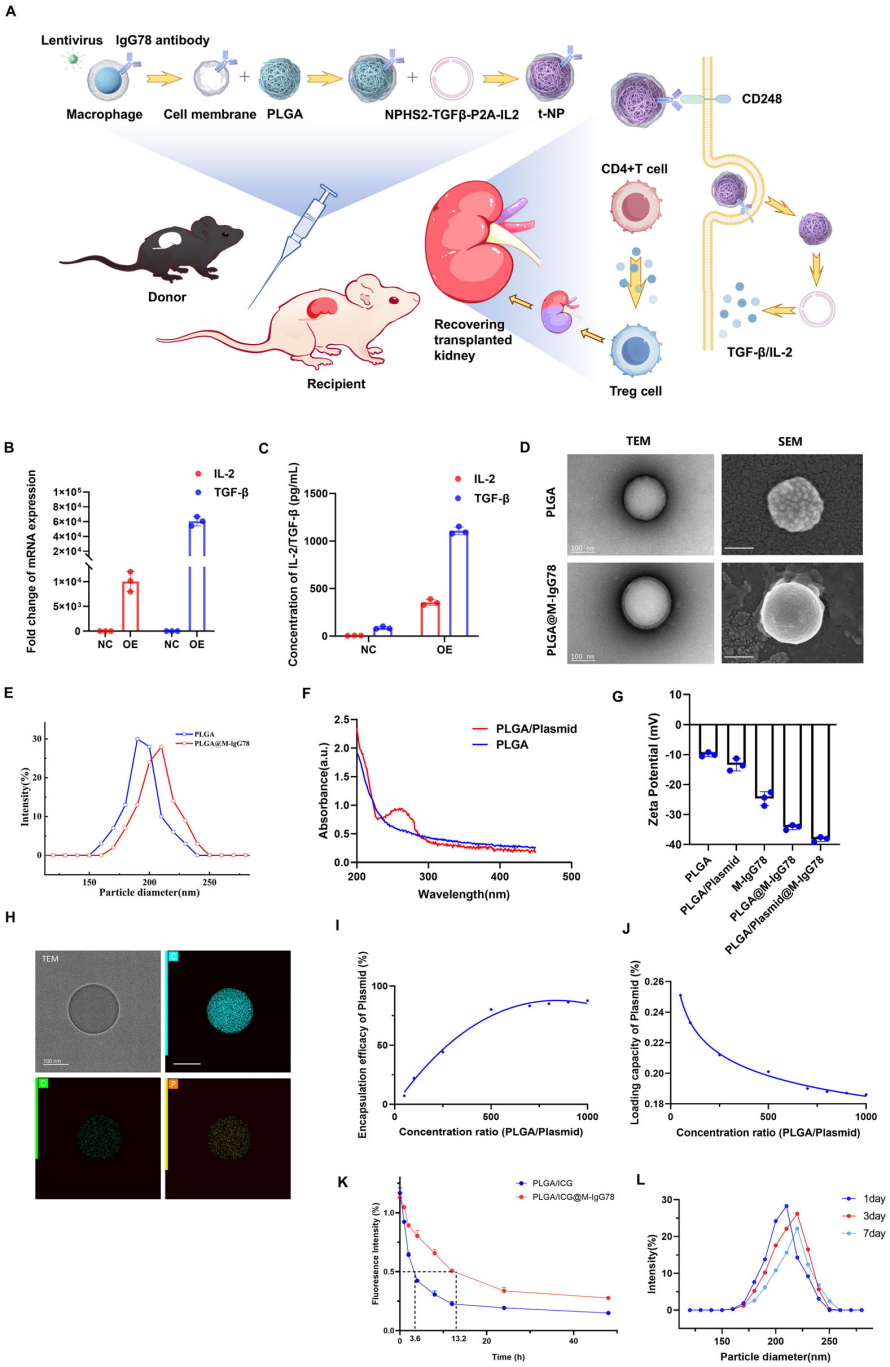
Fabrication and characterization of membrane‐coated nanoparticles.A) Schematic of PLGA/Plasmid@M‐IgG78 nanoparticle preparation process and induced Treg cells from CD4^+^T cells by tNPs. B,C) qPCR and ELISA detection of IL‐2 and TGF‐β which was expressed in MES13 cells by plasmid. D) TEM and SEM images of PLGA and PLGA@M‐IgG78. Scale bar: 100 nm. E) Size distributions of PLGA and PLGA@M‐IgG78. F) Ultraviolet spectroscope of PLGA and PLGA@M‐IgG78. G) Zeta potentials of PLGA and PLGA@M‐IgG78. H) EDS analysis to show the position of C, N and P elements. I–J) Plasmid loading property of PLGA. K) Blood half‐life of PLGA/ICG and PLGA/ICG@M‐IgG78. L) Size distributions change of PLGA@M‐IgG78 over time.

To develop localized Treg expansion strategies for durable graft tolerance, we engineered Treg‐promoting nanoparticles (tNPs) encapsulating plasmid DNA constructs (**Figure** **2**A; Figure S3C, Supporting Information). Initial validation via qPCR and ELISA confirmed IL‐2 and TGF‐β co‐expression from transfected cells (Figure 2B,C).

For precision targeting, we designed lentiviral vectors encoding anti‐CD248 antibody (IgG78). Transduction of the RAW264.7 cell line and primary macrophages achieved stable surface IgG78 expression, verified through flow cytometric quantification and confocal imaging (Figure S3A,B, Supporting Information). Cell membrane extraction followed by sequential extrusion generated CD248‐targeting tNPs through macrophage‐derived membrane coating of PLGA cores (200 nm average diameter). Cryo‐EM and atomic force microscopy confirmed intact membrane‐protein orientation on nanoparticle surfaces (Figure 2D,E). Physicochemical characterization demonstrated efficient plasmid encapsulation via UV–vis spectral DNA signature retention, surface charge inversion (zeta potential), and elemental phosphorus mapping (EDS), confirming nucleic acid incorporation (Figure 2F–H). Optimization studies established 500:1 (PLGA:plasmid) mass ratio as optimal (Figure 2I,J).

In order to detect the half‐life of nanoparticles in vivo, we conducted in vivo experiments. The results showed that the cell membrane‐encapsulated nanoparticles significantly prolonged the half‐life in mice (Figure 2K). The stability test showed that the nanoparticles could maintain the stability of particle size and potential within 1 week under physiological conditions (Figure 2L; Figure S3C, Supporting Information). The above results indicate that we synthesized stable plasmid‐loaded nanoparticles.

Next, we tested the biosafety of tNPs. In vitro experiments showed that tNPs and low doses of sirolimus did not affect cells (Figure S4A,B, Supporting Information). We injected nanoparticles into healthy mice and collected samples for testing two weeks later. The liver function and organs of mice were not affected (Figure S4C,D, Supporting Information). In addition, the use of tNPs after renal transplantation in rats did not affect liver function (Figure S4E, Supporting Information).

### 2.3 Targeting Ability and Tissue Specificity of tNPs

**Figure 3:**
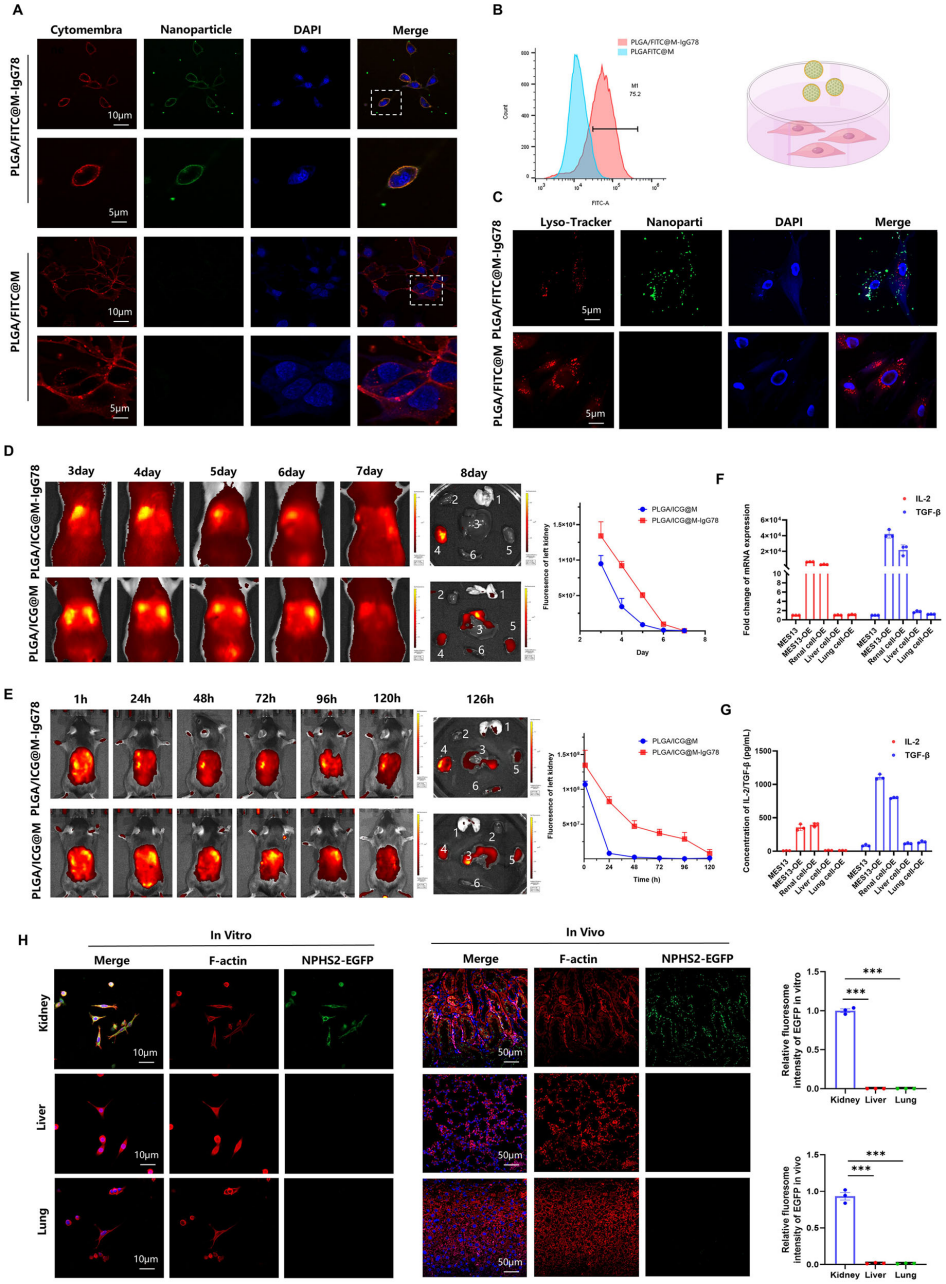
Targeting ability and tissue specificity of tNPs A) Representative IF staining to show nanoparticles(green) bending at cell membrane(red). Scale bars: 10 µm and 5 µm. B) Flow cytometry to show nanoparticles bending at cell membrane. C) Representative IF staining to show that part of nanoparticles(green) were obtained by lysosome(red) while the others were not. Scale bars: 5 µm. D) Transplanted kidney recognition capabilities of tNPs in transplanted Rats. Bioluminescence was noted in the images of two kidneys over 192 h after injection of tNPs. Sample size = 3. E) Injurited kidney recognition capabilities of tNPs in operated mice. Bioluminescence was noted in the images of two kidneys over 126 h after injection of tNPs. Sample size = 3. F–G) qPCR and ELISA detection of IL‐2 and TGF‐β which was expressed in primary kidney cells, lung cells, liver cells and MES‐13 cells by plasmid. H) Organ specificity of tNPs was detective by IF staining in transplanted Rats tissue and primary cells from different organs. Scale bars: 10 µm and 50 µm.

Following comprehensive tNP characterization, we evaluated their targeting specificity and biodistribution profiles. Flow cytometric quantification and IF imaging confirmed IgG78‐functionalized tNPs specifically bound renal stromal cells with subsequent receptor‐mediated endocytosis (**Figure** **3**A–C). Biodistribution profiling employed two preclinical models: rat renal transplantation – Orthotopic grafts received indocyanine green (ICG)‐loaded tNPs on postoperative day (POD) 1. In vivo fluorescence tracking revealed selective tNPs accumulation in allografts by POD3, with sustained retention (> 7 days) versus non‐targeted controls (Figure 3D). Murine renal ischemia‐reperfusion injury – Unilateral ischemic kidneys received immediate post‐ischemic tNPs administration. Spatial‐temporal analysis demonstrated injury‐specific tNPs localization within 24 h, persisting through POD7 (Figure 3E). This dual‐model validation establishes macrophage membrane‐displayed IgG78 as an effective renal targeting strategy, demonstrating both transplant‐specific and injury‐responsive delivery precision.

Capitalizing on the renal‐specific promoter (NPHS2) in our plasmid design, we systematically evaluated tNPs organotropism. In tri‐lineage comparative studies (renal vs hepatic vs pulmonary stromal cells), qPCR quantification, cytokine secretion profiling (ELISA), and subcellular localization analysis consistently demonstrated exclusive transgene expression in renal stromal populations (Figure 3F,G). In vivo validation through NPHS2‐EGFP delivery in rat transplant models revealed kidney‐restricted fluorescence signals upon confocal histopathology (Figure 3H). This dual‐targeting strategy – combining CD248‐directed homing with tissue‐specific promoter activity – effectively confined therapeutic effects to allografts while minimizing systemic exposure.

### 2.4 tNPs Induce Treg Cells In Vitro

**Figure 4:**
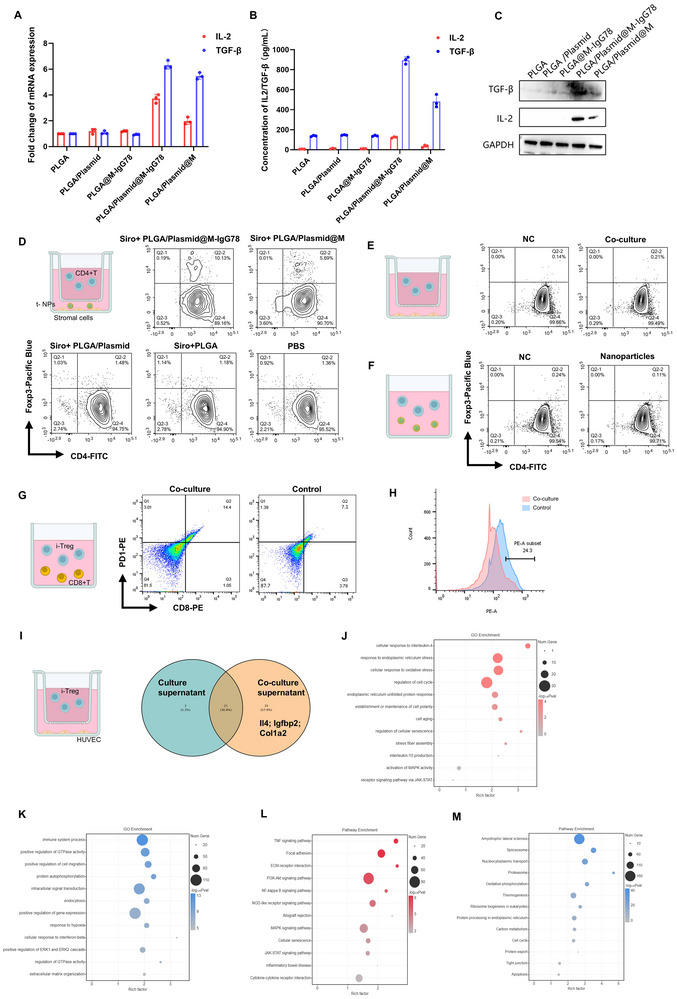
tNPs induce Treg cells in vitro. A–C) qPCR, ELISA and WB detection of IL‐2 and TGF‐β in co‐cultured system which include different nanoparticles and MES‐13 cells. D) Flow cytometry for detecting Treg cells in co‐cultured system which include different nanoparticles, CD4^+^T cells and MES‐13 cells. E) Flow cytometry for detecting Treg cells in co‐cultured system which include CD4+T cells and MES13 cells. F) Flow cytometry for detecting Treg cells in co‐cultured system which include tNPs and CD4^+^T cells. G) Flow cytometry to show the proportion of PD1^+^CD8^+^T cells in co‐culture system which include i‐Treg cells and CD8^+^T cells. H) After CFSE staining to CD8^+^T cells, we co‐culture i‐Treg and CD8^+^T cells. Flow cytometry to detect the fluorescence intensity of CD8^+^T cells. I) Proteomics detected the protein components in the co‐culture supernatant which include i‐Treg cells and HUVEC cells. J–M) GO and KEEG analysis of different genes with two groups.

Having established tNPs bioactivity, we systematically evaluated their Treg‐inducing capacity. Combinatorial analysis through qPCR, WB, and ELISA revealed only tNPs achieve therapeutic cytokine production (**Figure** **4**A–C). In our optimized CD4^+^ T cell‐stromal cell co‐culture system (pharmacologically relevant sirolimus concentrations preserving stromal viability; Figure S4B, Supporting Information), functional tNPs demonstrated dramatically enhanced Treg induction (CD4^+^Foxp3^+^) compared to single‐component formulations (Figure 4D–F). This induction exhibited time‐dependent enhancement (48to 96 h), mirroring canonical cytokine‐driven Treg differentiation patterns (Figure S5A,B, Supporting Information).

To investigate the functional characteristics of induced Treg cells (i‐Treg), we conducted a series of co‐culture experiments. First, i‐Treg cells demonstrated significant immunosuppressive effects when co‐cultured with CD8^+^ T cells, as evidenced by increased PD1^+^CD8^+^ exhausted T cell populations (Figure 4G) and suppressed T cell proliferation confirmed by CFSE staining (Figure 4H). Second, in co‐culture systems with endothelial cells (HUVECs), proteomic analysis of culture supernatants revealed substantial IL‐4 production by i‐Treg cells (Figure 4I). Transcriptomic profiling of the endothelial cells showed upregulation of HMGB1 and XBP1 (Figure S6A,B, Supporting Information), with subsequent pathway analysis demonstrating activation of IL‐4‐associated signaling cascades along with MAPK and AKT pathways (Figure 4J–M). Notably, these findings align with existing evidence suggesting IL‐4′s role in tissue repair.^[^ ^15^ ^]^ Besides, we detected the stability of i‐Treg cells (Figure S5C, Supporting Information). Collectively, our data confirm the successful in vitro induction of functional Treg cells using tNPs.

### 2.5 tNPs Induce Treg Cells In Vivo and Promote Local Immune Tolerance of Grafts

**Figure 5:**
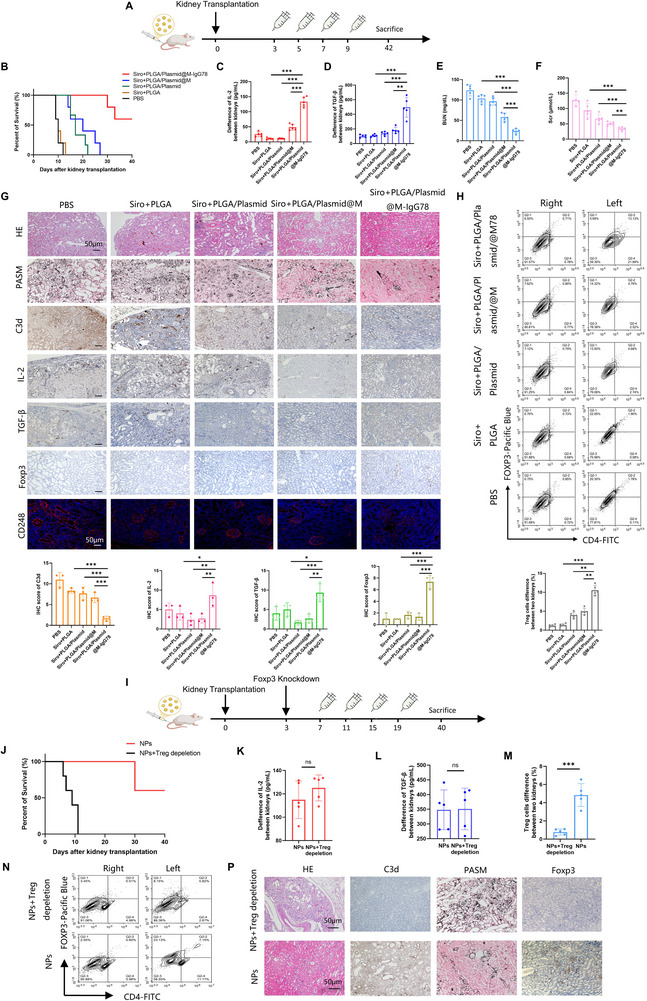
tNPs induce Treg cells in vivo and promote local immune tolerance of grafts. A) A schematic diagram of the tNPs treatment strategy in kidney transplantation model. B) The survival curve showed that tNPs prolonged the survival time of the receptor. Sample size = 5. C,D) The content of IL‐2 and TGF‐β of transplanted kidney was detected by ELISA. E,F) BUN and Scr content of receptors in day7. G) Representative images and statistical charts of IHC and IF staining for HE, PASM, C3d, IL‐2, TGF‐β, Foxp3 and CD248. Scale bars: 50 µm. H) The representative pictures and statistical charts of flow cytometry show that tNPs increased Treg cells. I) A schematic diagram of the tNPs treatment strategy with Foxp3 knockdown. J) The survival curve showed that knockdown of Foxp3 offsets the effect of tNPs. Sample size = 5. K,L) The content of IL‐2 and TGF‐β of transplanted kidney was detected by ELISA. M,N) The representative pictures and statistical charts of flow cytometry show that tNPs increased Treg cells. P) Representative images of IHC staining for HE, PASM, C3d and Foxp3. Scale bars: 50 µm.

To validate the therapeutic potential of Treg‐promoting nanoparticles (tNPs), we established a rat unilateral orthotopic kidney transplantation model using Wistar rats as donors and Sprague‐Dawley (SD) rats as recipients (**Figure** **5**A). tNPs treatment significantly prolonged allograft survival and successfully increased the content of IL‐2 and TGF‐β in grafts (Figure 5B–D). Renal function preservation was confirmed by maintained serum creatinine and blood urea nitrogen levels (Figure 5E,F). Histopathological analysis revealed preserved renal architecture with reduced complement C3d deposition and increased IL‐2/TGF‐β in tNP‐treated grafts (Figure 5G). Enhanced immune tolerance was demonstrated by elevated intragraft Treg cell frequencies (Figure 5H). To establish Treg dependence, Foxp3 expression was selectively inhibited using antisense oligonucleotide (ASO) pretreatment^[^ ^16^ ^]^ (Figure 5I). This ablation completely abrogated tNP‐mediated survival benefits (Figure 5J). While TGF‐β and IL‐2 levels remained elevated, Treg cell reconstitution was impaired in ASO‐treated recipients (Figure 5K–N). Concomitant histopathological exacerbation further confirmed Treg dependency (Figure 5P). These findings collectively demonstrate that tNPs prolong transplant survival through Treg‐mediated immune tolerance mechanisms.

### 2.6 tNPs Protect the Renal Grafts After Ischemia‐Reperfusion Injury

**Figure 6:**
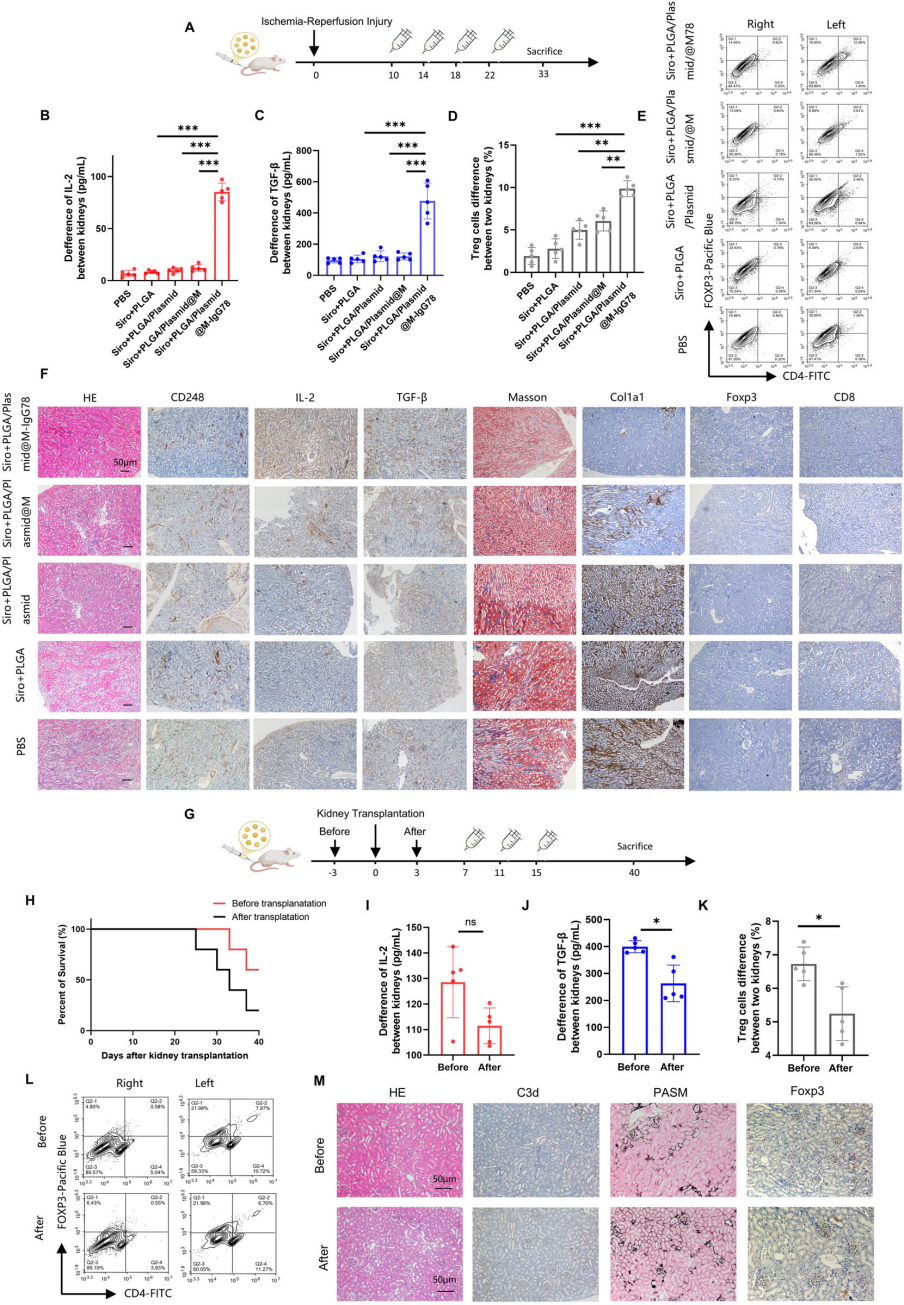
tNPs protect the renal grafts after ischemia‐reperfusion injury. A) A schematic diagram of the tNPs treatment strategy in ischemia‐reperfusion injury model. B,C) The content of IL‐2 and TG‐Fβ of injury kidney was detected by ELISA. Sample size = 5. D–E) The representative pictures and statistical charts of flow cytometry show that tNPs increased Treg cells. F) Representative images of IHC staining for HE, IL‐2, TGF‐β, Foxp3, Masson, Col1a1 and CD248. Scale bars: 50 µm. G) A schematic diagram of the tNPs treatment strategy in different time. H) The survival curve showed that tNPs were injected before surgery got better effects. Sample size = 5. I,J) The content of IL‐2 and TGF‐β of transplanted kidney was detected by ELISA. K,L) The representative pictures and statistical charts of flow cytometry show that tNPs increased Treg cells. M) Representative images of IHC staining for HE, PASM, C3d and Foxp3. Scale bars: 50 µm.

Our findings identify CD248 as a hypoxia‐responsive biomarker upregulated during the early post‐transplant phase. Building on the established dual role of Treg cells in immunosuppression and vascular repair during reperfusion injury,^[^ ^17^ ^]^ we investigated tNPs efficacy in renal ischemia‐reperfusion injury (IRI) using a murine renal IRI model (**Figure** **6**A). tNPs administration significantly enhanced intralesional IL‐2 and TGF‐β production, concomitant with Treg cell expansion in injured kidneys (Figure 6B–E). Histological assessment revealed a reduction in fibrotic area with robust Treg infiltration (Figure 6F), confirming renal protection during both IRI and rejection phases.

Mechanistically, we implemented a dual‐phase therapeutic regimen combining preoperative (3days before transplant surgery) and postoperative tNPs administration to recipients (Figure 6G). This strategic timing yielded superior outcomes, increasing allograft survival (Figure 6H). Preconditioned grafts exhibited higher Treg frequencies and sustained TGF‐β elevation (Figure 6I–L), correlating with preserved tissue architecture and reduction in complement C3d deposition (Figure 6M). Notably, these data establish CD248 as a clinically valuable biomarker enabling tNP‐mediated protection across two critical transplantation windows: early ischemia‐reperfusion injury (0–72 h post‐op) and subsequent adaptive immune rejection phase (day 7–14).

## 3 Discussion

While kidney transplantation has revolutionized end‐stage renal disease management, its clinical application remains constrained by critical organ shortages and immunosuppression‐related comorbidities including neoplasia, opportunistic infections, and calcineurin inhibitor nephrotoxicity.^[^ ^3^, ^18^ ^]^ The most direct and common side effects of immune system suppression are opportunistic infections of various pathogens. In severe cases, septic shock can be life‐threatening. However, when immunosuppressive agents are insufficient, the rejection of the graft may be aggravated. Due to the shortage of renal source, it is usually difficult for renal transplant patients to undergo a second renal transplantation. Therefore, how to balance the risk of immune rejection and infection has become a difficult problem for urologists.

Cell therapy and nanomedicine have become very promising therapies and are expected to partially replace immunosuppressive agents. Previous studies have tended to target the delivery of immunosuppressants to the transplanted kidney, thereby reducing the side effects of systemic medication. However, excessive immunosuppressive agents in the kidney aggravate kidney damage, which in turn leads to graft dysfunction. Macrophage membrane‐encapsulated nanoparticles have attracted much attention due to their good biomimetic properties. It can not only resist the phagocytosis of macrophages in the blood circulation, but also can directly deliver the medicine to the cells through the principle of membrane fusion. At the same time, without endocytosis, the plasmid in the nanoparticles can escape the degradation of lysosomes and play a role.

With regard to the diameter of nanoparticles, in the rejection model of renal transplantation, 200 nm diameter nanoparticles can also be delivered to the renal parenchyma due to the destruction of vascular structure caused by immune attack. Besides, the cell therapy of autologous transfusion of Treg cells is actively carried out in the field of renal transplantation. Clinical trials have shown that cell therapy reduces the risk of infection, but does not improve the long‐term survival of renal transplant patients, compared with the use of conventional immunosuppressants. This is partly due to the fact that Treg cells expanded in vitro cannot survive for a long time in vivo, and Treg cells used throughout the body cannot be enriched in grafts. Therefore, how to induce organ‐specific immune tolerance is an urgent problem to be solved.

Our nanotherapeutic platform addresses these issues through spatially controlled immunomodulation. The developed dual‐targeting nanoparticle system achieves compartmentalized Treg cells expansion via stromal cell‐specific CD248 engagement and renal parenchyma‐restricted transgene expression (NPHS2 promoter). This spatial precision resolves two historical dilemmas: 1) systemic cytokine toxicity from traditional IL‐2/TGF‐β therapies, and 2) functional instability of adoptively transferred Tregs (≤40% survival at 1 month post‐infusion^[^ ^19^ ^]^). Crucially, localized Treg polarization preserved antimicrobial immunity in treated animals—a critical limitation of current immunosuppressive cocktails.

Three paradigm‐shifting innovations emerge from this work: First, our single‐cell atlas‐driven approach identified CD248 as a mechanoresponsive stromal determinant in rejection, with HIF‐1α/IL‐1β co‐regulation explaining its microenvironmental specificity. Second, the biomimetic macrophage membrane coating synergizes with NPHS2‐driven expression to achieve higher renal accumulation vs non‐targeted nanoparticles. Third, beyond rejection suppression, we uncovered Treg‐mediated vascular repair—a pleiotropic mechanism reconciling historical contradictions about IL‐4 in transplantation.

IL‐4 is a Th2‐type cytokine generally considered to have anti‐inflammatory and immune‐regulatory effects. However, its role in kidney transplantation is complex. IL‐4 can promote the differentiation of B cells into plasma cells, which in turn produce donor‐specific antibodies (DSA), potentially increasing the risk of rejection.^[^ ^20^ ^]^ Nevertheless, IL‐4 also has potential in inducing immune tolerance. Some studies have shown that IL‐4 can promote the differentiation and function of Treg cells,^[^ ^21^ ^]^ inhibit Th1‐type immune responses, and reduce the occurrence of acute rejection. Additionally, IL‐4 may regulate the function of dendritic cells (DC) to promote long‐term graft survival.^[^ ^22^ ^]^ IL‐4 also promotes angiogenesis and remodeling.^[^ ^15^ ^]^ In our study, we found that IL4 activates multiple signaling pathways in endothelial cells, participating in the vascular repair process. In summary, the specific role of IL4 post‐kidney transplantation warrants further exploration, but our work further emphasizes the role of this cytokine in the post‐transplant period.

Inevitably, this study still has certain limitations. First, the current experiments are mainly based on rodent models, whose immune microenvironments differ from those of humans, necessitating further validation in non‐human primates. Long‐term treatment experiments on large animals might yield more convincing data. Second, the long‐term biosafety of the nanoparticles (such as metabolic pathways and potential immunogenicity) has not been fully clarified and requires systematic assessment of chronic toxicity. Additionally, clinical translation needs to address issues such as large‐scale production processes, cost control, and optimization of individualized dosing regimens. Future research could explore the combination of this strategy with other immunomodulatory therapies (such as checkpoint agonists or CAR‐Treg technology) to further enhance therapeutic efficacy.

In conclusion, this study provides a new method for inducing immune tolerance after kidney transplantation, which is both precise and safe. It is expected to promote a paradigm shift in clinical practice from “broad‐spectrum immunosuppression” to “local immune modulation,” paving a new way for patients with end‐stage renal disease to achieve long‐term graft survival and improved quality of life.

## 4 Experimental Section

### Bioinformatics Analysis

Single‐cell sequencing data was integrated from 11 kidney transplant patients from three independent datasets, classified according to clinical features (normal, rejection, and no‐rejection groups). Data come from Gene Expression Omnibus: GSE151671, GSE145927, and GSE131685. The total number of cells was 91 500 (Table S2, Supporting Information). Data preprocessing was performed using Seurat v4.0 for normalization, dimensionality reduction (principal component analysis, PCA), and clustering (resolution = 0.8) to define cell subpopulations. Differential gene analysis was conducted using the Wilcoxon rank‐sum test, with significantly different genes (*p*<0.05, log2 fold change >1) used for subsequent enrichment analysis. Intercellular communication networks were constructed using the CellChat package to analyze interactions between stromal cells and other cell types.

### Fabrication and Characterization of Nanoparticles

Plasmid construction and validation: The IL‐2 and TGF‐β genes were cloned into the pUC57 plasmid vector and transfected into MES‐13 cells using Lipofectamine 3000. After 48 h, cytokine expression was detected by qPCR (SYBR Green method) and ELISA (R&D Systems kit). Plasmid sequence is in Supporting Information.

Membrane‐coated nanoparticle fabrication: RAW264.7 cells and primary macrophages were transfected with a lentivirus system (IgG78 antibody gene). Antibody expression was verified by flow cytometry (FACS Calibur) and immunofluorescence.

Cell membranes of M‐IgG78 were collected according to a previously published protocol.^[^ ^23^ ^]^ In brief, 1 × 10^7^ transfected cells were washed with PBS three times and resuspended in 1 mL of hypotonic lysing buffer, followed by ultrasonic fragmentation. The homogenized solution was centrifuged at 3500 g for 5 min at 4 °C, and the supernatant was centrifuged again at 16 900 g for 30 min at 4 °C. The precipitates were washed with DNase‐ and RNase‐free water and stored at −80 °C for subsequent analysis.

1 mg PLGA and 2 µg Plasmid was mixed in 5 mL of deionized water to achieve the approximate drug concentration ratio (PLGA:plasmid = 500:1). After 3 min of ultrasonication, the mixture was stirred at room temperature for 4 h in a dark, nitrogen‐free environment. The product was collected via centrifugation at 12 000 rpm for 10 min and washed with deionized water three times.

Next, 200 µg of PLGA/Plasmid and 400 µg of M‐IgG78 were mixed in 1 mL of deionized water. After 5 min of ultrasonication in an ice bath, the mixture was extruded using Avanti Polar Lipids with 400 and 200 nm filter membranes (Whatman) successively.^[^ ^24^ ^]^ Membrane fusion was facilitated using a 10 × continuous extrusion (Figure S3E, Supporting Information).

The morphology of the nanoparticles was characterized by scanning electron microscopy (SEM, Hitachi SU8010) and transmission electron microscopy (TEM, JEOL JEM‐1400). The zeta potential (Malvern Zetasizer) and UV spectrophotometry (NanoDrop) were used to evaluate drug loading efficiency. Cell toxicity was assessed using the CCK‐8 kit at concentration gradients (0–100 µg mL^−1^) for 2 h.

In order to detect the stability of the nanoparticles, we dispersed the synthesized nanoparticles in normal saline, and regularly detected their particle size and Zeta potential within a week.

### Targeting and Organ‐Specificity Verification

In vitro experiments: First, nanoparticles loaded with the FITC fluorescent dye were constructed. The cell membrane was stained using a Dil staining kit (C1415S/Beyotime). Then, the two were co‐incubated for 30 min, followed by washing and fixation for microscopic observation to examine the binding of nanoparticles to the cell membrane. Similarly, lysosomes were labeled using a Lyso Tracker kit. In this case, the nanoparticles were co‐incubated with the cells for 2 h before microscopic observation to detect the entry of nanoparticles into the cells. Targeting efficiency was also detected by flow cytometry. Nanoparticles were co‐incubated with primary stromal cells from kidney, liver, and lung tissues to detected tissue specificity in a similar ways.

In vivo experiments: Kidney transplantation model: SD rats received orthotopic left kidney transplantation from Wistar rats, and ICG‐labeled nanoparticles (dose: 5 mg kg^−1^) were injected via the tail vein on postoperative day 1. The distribution of nanoparticles in the transplanted kidney was monitored by a small animal in vivo imaging system (IVIS Spectrum) at time points from postoperative day 1 to day 7.

Ischemia‐reperfusion model: C57BL/6 mice underwent 45 min of left kidney ischemia followed by reperfusion, with ICG nanoparticles injected immediately after reperfusion. Imaging was observed from 24 h to 7 days.

Organ‐specific expression: NPHS2 promoter‐driven EGFP expression was used, and kidney‐specific expression was detected by fluorescence microscopy (Olympus BX53) and ELISA.

### In Vitro Treg Cell Induction Experiment

A transwell co‐culture system was constructed between CD4^+^T cells (sorted by magnetic beads, Biolegend MojoSort) and renal stromal cells, with the addition of nanoparticles (10 µg mL^−1^) and low‐dose sirolimus (1 nM). We also used TGFβ (3 ng mL^−1^), IL2 (300 IU mL^−1^), and rapamycin (100 ng mL^−1^) to induce Treg cells.^[^ ^25^ ^]^ The proportion of Treg cells (CD4⁺Foxp3⁺) was detected by flow cytometry (anti‐CD4‐FITC, anti‐Foxp3‐PacificBlue antibodies, anti‐Foxp3‐FITC antibodies, BioLegend). In order to detect the stability of i‐Treg cells, we isolated the Treg cells induced by tNPs and cultured them under standard conditions (5% fetal bovine serum, 5% CO_2_) for 72 h to observe the expression of Foxp3.

In functional validation, i‐Treg cells were co‐cultured with HUVEC for 48 h, and the supernatant was analyzed by Luminex multiplex detection (Millipore). Endothelial cell transcriptome sequencing (Illumina NovaSeq 6 000) was performed to analyze differentially expressed genes (DESeq2, p <0.05). The RNA‐seq data are available in the GEO database (https://www.ncbi.nlm.nih.gov/bioproject/PRJNA1268035).

### In Vivo Efficacy Evaluation

Kidney transplantation model: SD rats were randomly divided into control (PBS) and nanoparticle treatment groups (5 mg kg^−1^, every other day, four times in total). Sirolimus was used in four groups (0.5 mg kg^−1^, every other day, two times in total). A low dose of sirolimus must be administered to get through the first 3 days of the hyperacute rejection phase, especially for transplantation between different strains of rats (Wistar to SD rats).

Survival was analyzed using the Kaplan‐Meier method, with differences analyzed by the Log‐rank test. Serum creatinine (Creatinine Assay Kit, Abcam) and blood urea nitrogen (BUN Kit, Sigma) were measured on postoperative days 7, 14, and 28. Transplanted kidney tissues were fixed in 4% paraformaldehyde, embedded in paraffin, and stained with H&E, C3d immunohistochemistry (anti‐C3d antibody, Abcam), and Treg cell counting (anti‐Foxp3 antibody, Cell Signaling Technology). The expressions of the marker were assessed according to the percentage and intensity of the IHC staining.

Treg dependency verification: Foxp3 ASO (50 µg per rat, twice weekly) was injected via the tail vein to inhibit Treg cell function, and the experiments were repeated. The antisense oligonucleotide sequence (ASO) targeting the rat FOXP3 gene was ATATGTATAGCTGGTT.

### Ischemia‐Reperfusion Injury Protection Experiment

C57BL/6 mice were randomly divided into control and treatment groups (nanoparticles 5 mg kg^−1^, injected 24 h preoperatively and immediately postoperatively). Kidney tissues were collected 24 h postoperatively to detect IL2, TGFβ (ELISA), and Treg cell proportion (flow cytometry). Masson's trichrome staining was used to assess fibrosis, with collagen deposition area quantified by ImageJ. The animal experiments in this study were approved by the Laboratory Animal Welfare and Ethics Committee of Fourth Military Medical University (No. 251 319).

### Detection of Circulation Half‐Life of Nanoparticles In Vivo

We injected 5 mg mL^−1^ tNPs into normal mice at a dose of 4 µL per gram of body weight via the tail vein. Blood (20 µL) was taken from the tail of the anesthetized mice at specific times after injection, diluted with an equal volume of PBS, and the fluorescence intensity of ICG in the blood was detected using a multifunctional microplate reader.

### Organ Toxicity Evaluation

After 2 weeks of PBS, PLGA/Plasmid, PLGA/Plasmid@M, or PLGA/Plasmid@M‐IgG78 injection, the mice were sacrificed through neck dislocation, and blood samples were obtained from the left ventricle of the heart. Next, 200 µL of serum was obtained from the collected blood samples through centrifugation at 3000 rpm and 4 °C for 10 min. Serum biochemical markers including alanine aminotransferase (ALT), aspartate transaminase (AST), blood urea nitrogen (BUN), serum creatinine (Scr) were detected using commercial kits and a multifunctional biochemistry analyzer (AU600; Olympus), according to the manufacturers’ instructions.

Moreover, the major organs—the heart, lungs, liver, kidneys, and spleen—of mice injected with PBS, PLGA/Plasmid, PLGA/Plasmid@M, or PLGA/Plasmid@M‐IgG78 were resected and fixed with 4% paraformaldehyde. After they were embedded in paraffin, the tissues were sliced in 4 µm‐thick sections and then subjected to H&E staining. The digital images of the stained sections were acquired using an inverted microscope (TE2000‐S; Nikon).

### Western Blot and qPCR Analysis

To obtain protein samples, cells were lysed in RIPA buffer. Equal amounts of protein samples were separated through 15% sodium dodecyl sulfate polyacrylamide gel electrophoresis and then transferred into polyvinylidene fluoride membranes. After they were blocked, the membranes were then incubated with appropriate primary antibodies(ab32142, ab308433, ab204914/abcam) and HRP‐conjugated secondary antibodies. The blots were visualized by using an electrochemiluminescence system. The α subunit and β subunit inhibitors of P38 were SB203580.

Total RNA was extracted from the isolated tissues by using Trizol, according to the manufacturer's protocol. cDNA synthesis was performed using the first‐strand cDNA synthesis kit. The relative gene expression was analyzed using TB green premix. The target mRNA expression was normalized to GAPDH.

### Data Sharing Statement

Anonymous participant data is available upon request with publication. The datasets generated and/or analyzed in this study are available in the National Center for Biotechnology Information Gene Expression Omnibus (PRJNA1268035).

### Statistical Analysis

Data are presented as mean ± standard deviation. Group comparisons were performed using t‐tests or one‐way ANOVA, with survival analysis conducted using the Log‐rank test (GraphPad Prism 9.0). A *p*‐value of <0.05 was considered statistically significant.

## Conflict of Interest

The authors declare no conflict of interest.

## Supporting information



Supporting Information

## Data Availability

The data that support the findings of this study are openly available in Gene Expression Omnibus at https://www.ncbi.nlm.nih.gov/geo/, reference number GSE151671, GSE145927 and GSE131685. These data were derived from the following resources available in the public domain: the National Center for Biotechnology Information Gene Expression Omnibus (PRJNA1268035).
