# Supplementary material for: Targeted Expansion of Treg Cells to Induce Immune Tolerance after Kidney Transplantation
Source: Adv Sci (Weinh). 2025 Oct 24;13(1):e07943. doi: 10.1002/advs.202507943 (PMC12767053; doi:10.1002/advs.202507943)
Supplement: Supplementary file 1 — Supporting Information [file ADVS-13-e07943-s001.docx]

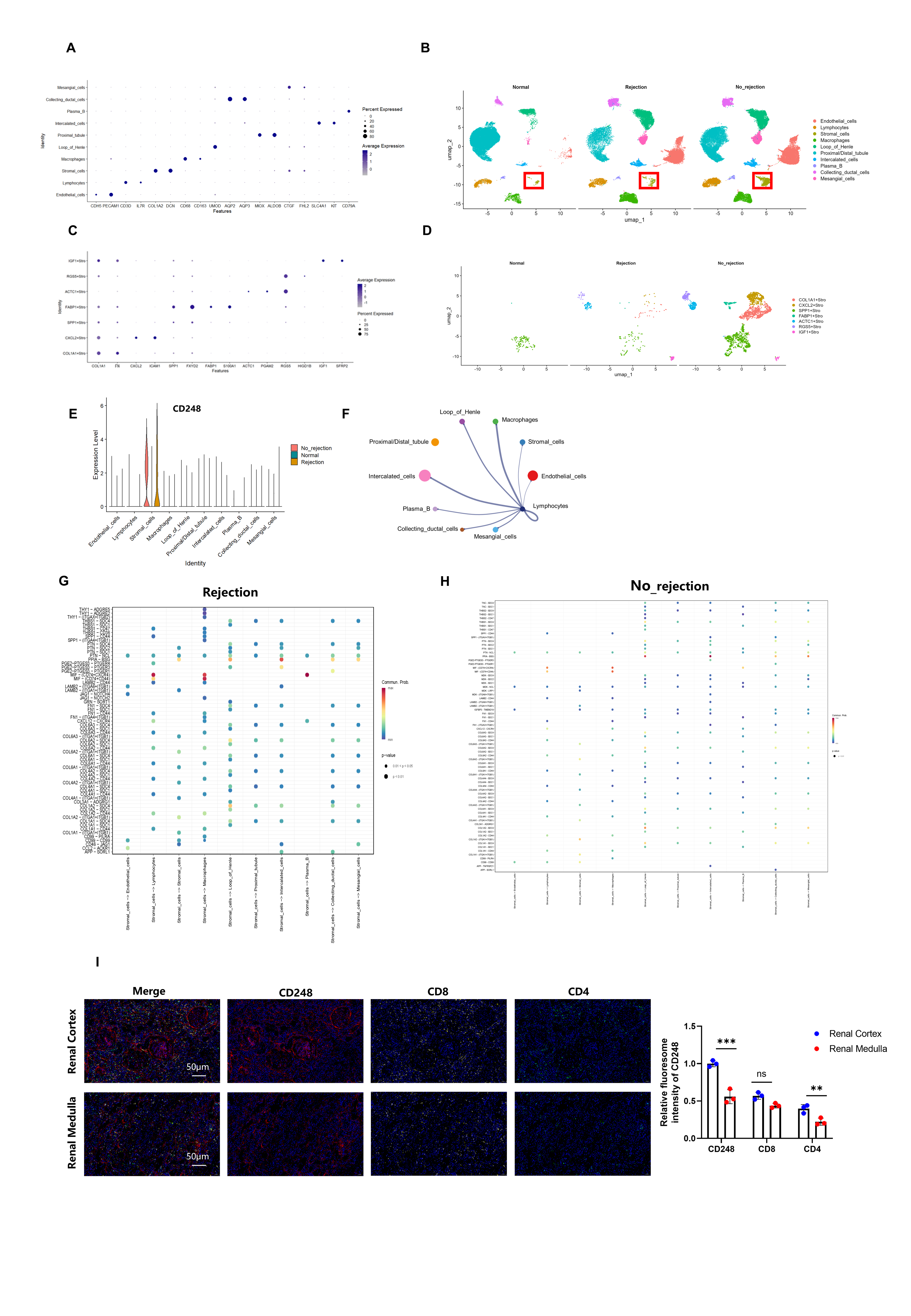


Supplementary Figure 1: CD248 of stromal cells was up-regulated after renal transplantation.

(A) Marker genes of all cells. (B) Umap image to show all cells in different group. The red box marked the stromal cells. (C)Marker genes of all stromal cells. (D) Umap image to show all stromal cells in different group. (E) Expression of CD248 divided by different group in all cells. (F) Cell chat between lymphocytes and other cells. (G-H) Cell chat between stromal cells and other cells in rejection and no rejection group. (I) IF staining and quantity analysis of CD248, CD8 and CD4 in renal cortex and medulla. Scale bars: 50μm.


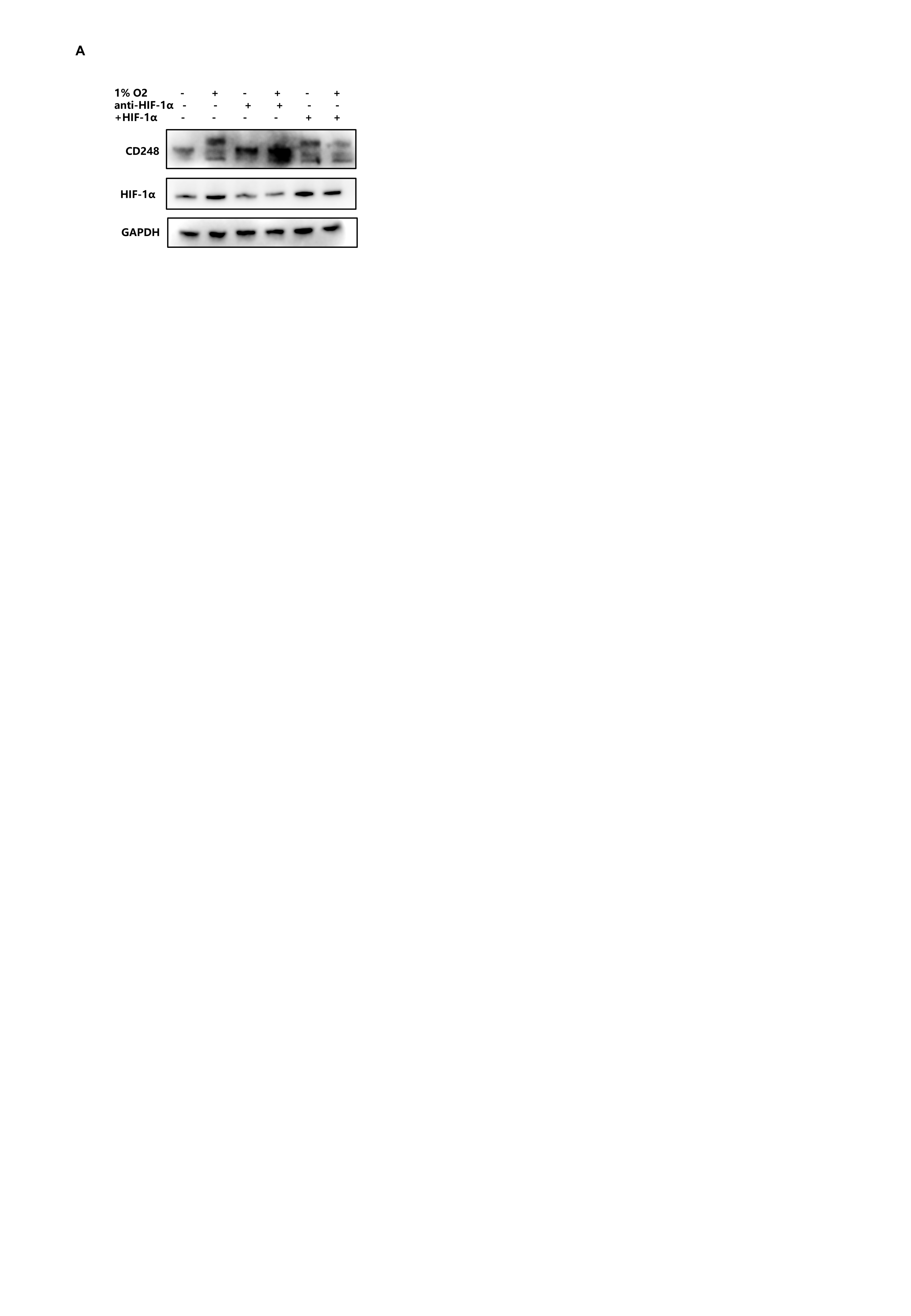


Supplementary Figure 2: Western blot to show HIF1α-CD248 axis in MES-13 cell line (A).


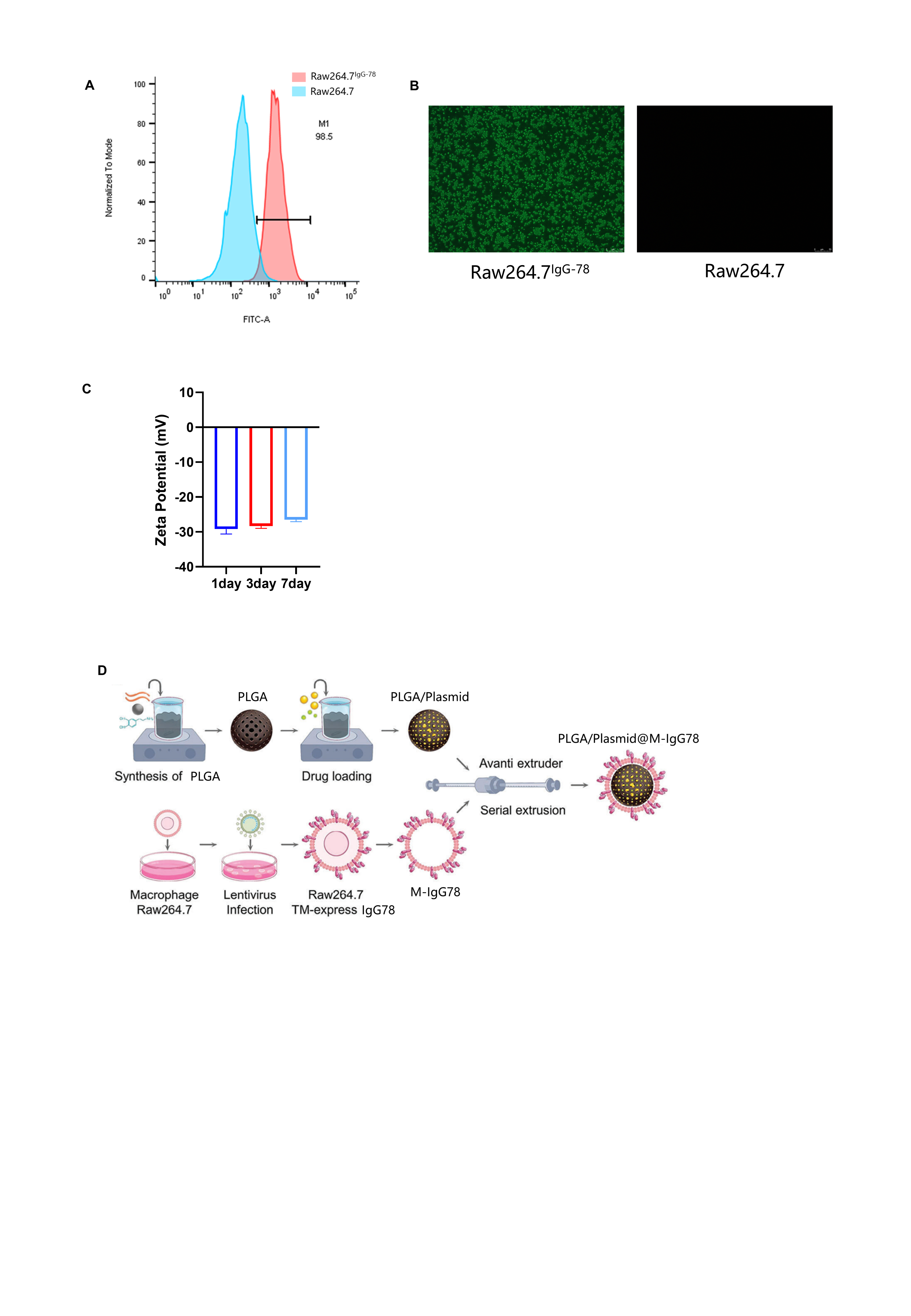


Supplementary Figure 3: Preparation for the synthesis of nanoparticles

(A-B) Flow cytometry and IF staining show that macrophages were infected by lentivirus and expressed fluorescence. (C) Zeta potential of tNPs changed from 1 day to 14 day. (D) A schematic diagram of the synthesis process of nanoparticles.


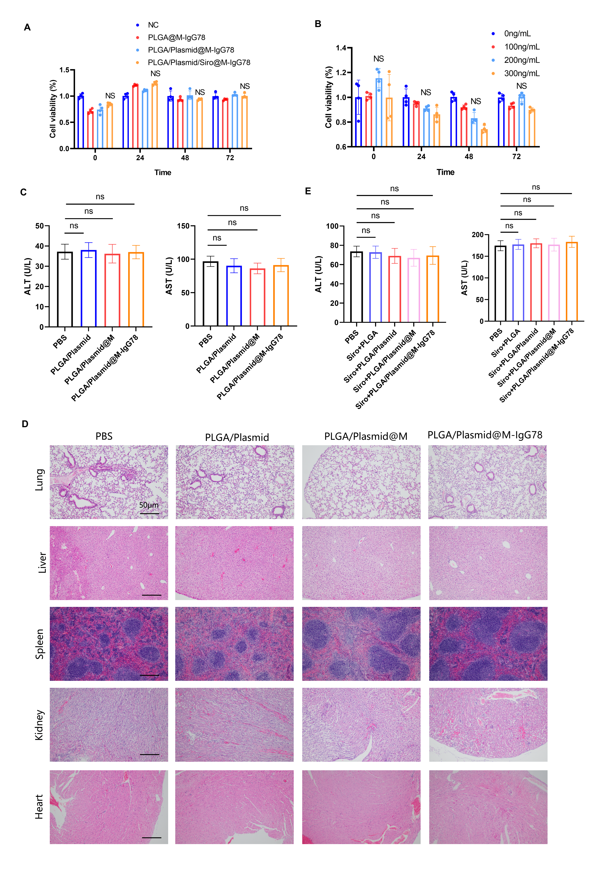


Supplementary Figure 4: Biosafety detection

(A) CCK8 to detect toxicity of nanoparticles components. (B) CCK8 to detect the toxicity of sirolimus in different concentration. (C) tNPs were injected into mouse for 2 weeks. ALT and AST detection of serum. Sample size=3. (D) HE staining of lung, liver, spleen, kidney and heart. Scale bars: 50μm. (E) tNPs were injected into Rat for 2 weeks. ALT and AST detection of serum. Sample size=3.


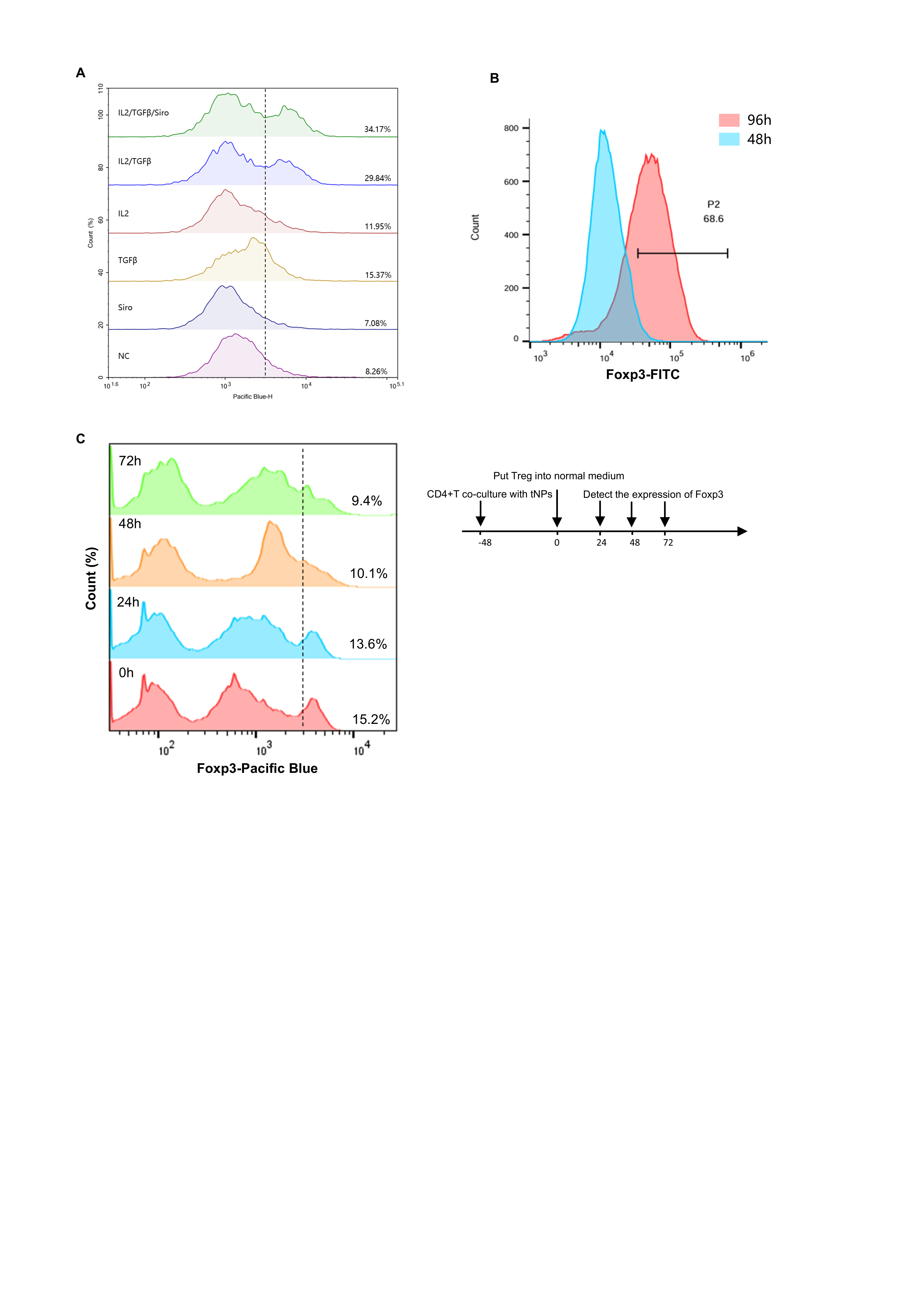


Supplementary Figure 5: i-Treg cell induction protocol

(A) Flow cytometry to show IL-2 and TGF-β combination induced Treg cells successfully. The effect of using cytokines alone is not good. (B) Prolonging the stimulation time of TGF-β and IL-2 can further improve the induction efficiency. (C) Stability test of i-Treg over 72 hours.


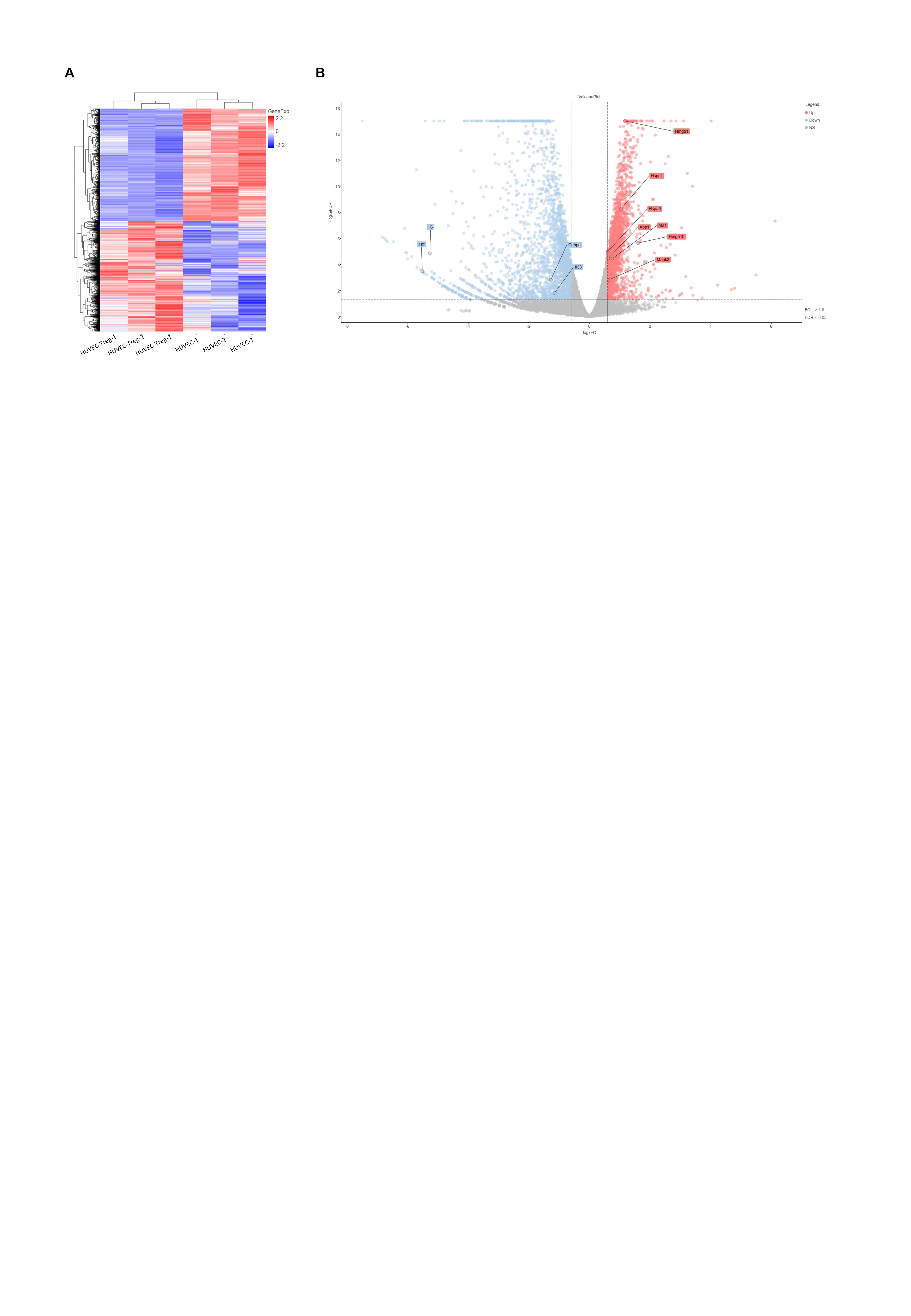


Supplementary Figure 6: RNA sequencing for HUVEC

(A) Heat map show the different genes between HUVEC-Treg group and HUVEC group. (B) RNA-seq for co-culture group and control group which reveals the influence from i-Treg cells to endothelial cells.

Supplementary Table 1: Allogeneic renal transplantation patient information

| Patient number | age | sex | Post operation day |
| --- | --- | --- | --- |
| 1 | 40 | Male | 33 |
| 2 | 74 | Male | 105 |
| 3 | 60 | Male | 472 |
| 4 | 55 | Female | Clear cell renal carcinoma  Paracancerous tissues were used as normal controls. |

Supplementary Table 2: Single-cell sequencing data information

| datasets | Sample name | Greoup | Endothelial_cells | Lymphocytes | Stromal_cells | Macrophages | Loop_of_Henle | Proximal/Distal_tubule | Intercalated_cells | Plasma_B | Collecting_ductal_cells | Mesangial_cells | Sum |
| --- | --- | --- | --- | --- | --- | --- | --- | --- | --- | --- | --- | --- | --- |
| GSE145927 | day11 | rejection | 1204 | 171 | 66 | 1215 | 160 | 1523 | 124 | 23 | 65 | 28 | 4579 |
|  | day232 | rejection | 1821 | 55 | 40 | 264 | 2222 | 1357 | 444 | 29 | 270 | 162 | 6664 |
|  | day2542 | rejection | 6426 | 125 | 17 | 832 | 2629 | 468 | 752 | 24 | 1198 | 1722 | 14193 |
|  | day28 | no rejection | 5900 | 426 | 803 | 515 | 3225 | 3677 | 434 | 142 | 1050 | 1591 | 17763 |
|  | day5 | no rejection | 2802 | 1076 | 1595 | 1335 | 2066 | 6496 | 796 | 122 | 1314 | 217 | 17819 |
| GSE131685 | GSM204 | normal | 7 | 542 | 0 | 224 | 16 | 5320 | 19 | 90 | 2 | 2 | 6222 |
|  | GSM205 | normal | 4 | 147 | 0 | 78 | 133 | 4195 | 6 | 10 | 2 | 0 | 4575 |
|  | GSM206 | normal | 7 | 189 | 2 | 415 | 177 | 9436 | 37 | 30 | 78 | 8 | 10379 |
| GSE151671 | GSM971 | normal | 302 | 45 | 111 | 69 | 1494 | 2003 | 89 | 16 | 382 | 107 | 4618 |
|  | GSM972 | rejection | 82 | 593 | 143 | 257 | 55 | 767 | 45 | 38 | 41 | 17 | 2038 |
|  | GSM973 | no rejection | 100 | 980 | 132 | 527 | 61 | 739 | 42 | 90 | 21 | 8 | 2700 |
|  | Sum |  | 18655 | 4349 | 2909 | 5731 | 12238 | 35981 | 2788 | 614 | 4423 | 3862 | 91550 |

Supplementary plasmid sequence:

1. NPHS2 sequence:

GCCCTCCTATTTAGTCTCTCTGCCACCTACAAATTGAGAAAGTCAAATTTAGTAAGTCCTTATAGTTTCCAGCTCTAAAACAACAGGATTTGTAACATCATGTAGTGCCAATCAATTCCAGCCTTTCCATTAAATAATCCAAATGAATCAGTATTCATGCATTCACTTATTCATATGGATATAATACTTCAAAACACTAACTGGGAATAAGTGTAATACTTTAGAAAGCCTTGCACTCTTTCATAATCTTTCACCTTGGAAACATAAAAGTAAACAGCTTTAATTGATCCTCCCTTTCTTCTAGTAACCCAGTTCCCACAGATGTCAGTTTCTTAACAAATCCAAACAAGGAGTAAAGTATTGAGAGTTTCAGAATGTGTGCATATCATATTTTCAGCAAAGGGATGAGCAACACTTTCAATTAAAAAGGAATCTAAAAGAAAACAGCAGCATCCTTGTAAAAGTAGATGAGGACAGCCAGCTGGACTTAGCTGCTAACTCAGCACTCCCTTCCCCAACTCCTCCACCTTCCAGCCATGCTGCATTGACCTTTTTATCATGGAATGGTATGCAGCTTCTCTGATTTTGTCATTGTCACTCATAATCCCAGCAGACTTGTGTTATAAGCAGCTGCTTATATTGCTGTTATGTATAGATATAGAAATGTAACTTGTCAGTGGTCAAAGAAGCCAAGGAAACTGAGCAAAGGTGACTGAGAACAAGTGCTCAGCATGGGCTCCAACACCCAGTAGGGCCTTTTCTCATTGATTTTTCTCCTACAAGGAATTTCTCTCTTATTGAAAGACAGTACAGTTTGGGGAATATCATTTGACCAGATATCCTGCTCCATTCTTGGAATGAAAAACAGGTGCAATTTTAGTATAAGAGTCTAGCTAGGGATGCGGATGAAATTAATTTATAACAGGACCAGGCTGAGGAAAAACACCAATAAATGCTACAAATAGTTTGTCACTTTTTCTAACAGGGAAATCTCTAAAGGATGATAAAGATGCTAGCTGTGTTTAATAGCAAATGTTAATTTGGGGGAATATGAAATAATCATGATATCTTTCTCTCTTTGAGCCAAACGCAGCTTTGGAATTTTTCTCACTGTAGTGTTGTAGGCAGAAACTATCAACTGAGGCAGGCCCTTGCGAGGAAATATTTATCCTCCTTAGCCCATGCAATGATCAGGTTTAGACAGTAAAAAATCTATTTAAGTGACTGGATTGCTCATCTTTTATTTACTTTACTCCCAGTGGCCTAACCTGGAACATATAGAAGAAAGTAGTGGAATGGTTTTTCCAGACGCACTTCAGTTACTTCAGGTCCTCAGTGTTTAATAAGGTTTTGGGAGAGAAAAAAAGCTATTGCTGTGTGAATTCAATAAATATTTAAAAAATTTAAAAAATCAGTGAGCATTATTTCTGCCATCATGTGATCTGAACTATCTGAGTAGTATCATAAATCTTGGGTGATGGGTTACCTTGACCAACCTCCCTTTCAGTAAGAAACAAATTATTAACAGAAAGTGAAAGAGAAATAAACCTATTCAATGTTTCAAGTCCCTCAGAAGAGGGTGAGGCAGCTCCCCAGTTCGTTGCTAGATCCAGCCTGGCCAATGAACCCTGAAAAAGCCCAACTCCTGCTTTCATCATGGAAGCACGGGACAAAGTGTCTCTTCCTAAAAACAGAAGTTAGACCAGACCCCTTCCTGCCTATGATTCTTCAAGAAGCATTGCATCATCAACATCAGGCATAAGCATTAATAAAGACCCTAAATAATAACAGAGATGAAACACATCGCAAAGAGAGTTTTCTTTTATCCCCTTTAAAATGTAAATACTCCCAGGAGGAATCAGCCAACATCATTAGGGGTTAATGCATATGTAGAATAACTAGGGCCAGGATATAAAATAAGAAATATGTAGGGAGGAGAGAAAGGCATCCTTGAGACGACTCCAAGAAGGAAAGTTGGGGATGAGGCGAAATTTCTGATTTTACCTTAAAGTGACCCTAATTCGATGACCTTTTGTGGTTTTTTTCTTTTTTCTTTTTTACTTGGCCCTGCCCAAGCAGGACCTAAAAACAAACAGACAAAAAAGGTTACTAACAACTGTTCCTCTCCACGAAAATCTGCAGTAAAAGGTAAAAGATGTATTCGTTTTGAAGAGAAACCAGAGCTTGCGATGAGCTTCTGTATCTCCGTCAGCCCTCTAGCATGACATTAGGAACCCTCCAGGAGATGAGTCTTCACAGCCCGGGTTGGCACCTGCAGACACGCACTTTTCAACGCCCGCACCCTGCCCGGGGCCGGCTCTCCCACCCAGGCCTCTCTCTGCTTCAGCGCCGCCCCGGCCGTGGGAGTCGGCGGGCGCAGTCCACAGCTCCACCAAGACACAGCTGTCGGGGTTCCGGGTGCGCCCCGCCCGCGGCCCCGGTGTCCCGCCCCTCGCCCTCAGCCCCCACCCGACGGTCTTTAGGGTCCCCCGGGCACGCCACGCGGACCCGCAGCGACTCCACAGGGACTGCGCTCCCGTGCCCCTAGCGCTCCCGCGCTGCTGCTCCAGCCGCCCGGCAGCTCTGAGG

2. TGF-β sequence:

ATGCCGCCCTCGGGGCTGCGGCTACTGCCGCTTCTGCTCCCACTCCCGTGGCTTCTAGTGCTGACGCCCGGGAGGCCAGCCGCGGGACTCTCCACCTGCAAGACCATCGACATGGAGCTGGTGAAACGGAAGCGCATCGAAGCCATCCGTGGCCAGATCCTGTCCAAACTAAGGCTCGCCAGTCCCCCAAGCCAGGGGGAGGTACCGCCCGGCCCGCTGCCCGAGGCGGTGCTCGCTTTGTACAACAGCACCCGCGACCGGGTGGCAGGCGAGAGCGCCGACCCAGAGCCGGAGCCCGAAGCGGACTACTATGCTAAAGAGGTCACCCGCGTGCTAATGGTGGACCGCAACAACGCCATCTATGAGAAAACCAAAGACATCTCACACAGTATATATATGTTCTTCAATACGTCAGACATTCGGGAAGCAGTGCCCGAACCCCCATTGCTGTCCCGTGCAGAGCTGCGCTTGCAGAGATTAAAATCAAGTGTGGAGCAACATGTGGAACTCTACCAGAAATATAGCAACAATTCCTGGCGTTACCTTGGTAACCGGCTGCTGACCCCCACTGATACGCCTGAGTGGCTGTCTTTTGACGTCACTGGAGTTGTACGGCAGTGGCTGAACCAAGGAGACGGAATACAGGGCTTTCGATTCAGCGCTCACTGCTCTTGTGACAGCAAAGATAACAAACTCCACGTGGAAATCAACGGGATCAGCCCCAAACGTCGGGGCGACCTGGGCACCATCCATGACATGAACCGGCCCTTCCTGCTCCTCATGGCCACCCCCCTGGAAAGGGCCCAGCACCTGCACAGCTCACGGCACCGGAGAGCCCTGGATACCAACTATTGCTTCAGCTCCACAGAGAAGAACTGCTGTGTGCGGCAGCTGTACATTGACTTTAGGAAGGACCTGGGTTGGAAGTGGATCCACGAGCCCAAGGGCTACCATGCCAACTTCTGTCTGGGACCCTGCCCCTATATTTGGAGCCTGGACACACAGTACAGCAAGGTCCTTGCCCTCTACAACCAACACAACCCGGGCGCTTCGGCGTCACCGTGCTGCGTGCCGCAGGCTTTGGAGCCACTGCCCATCGTCTACTACGTGGGTCGCAAGCCCAAGGTGGAGCAGTTGTCCAACATGATTGTGCGCTCCTGCAAGTGCAGC

3. P2A sequence:

GGAAGCGGAGCCACGAACTTCTCTCTGTTAAAGCAAGCAGGAGATGTTGAAGAAAACCCCGGGCCT

4. IL-2 sequence：

ATGTACAGCATGCAGCTCGCATCCTGTGTCACATTGACACTTGTGCTCCTTGTCAACAGCGCACCCACTTCAAGCTCCACTTCAAGCTCTACAGCGGAAGCACAGCAGCAGCAGCAGCAGCAGCAGCAGCAGCAGCAGCACCTGGAGCAGCTGTTGATGGACCTACAGGAGCTCCTGAGCAGGATGGAGAATTACAGGAACCTGAAACTCCCCAGGATGCTCACCTTCAAATTTTACTTGCCCAAGCAGGCCACAGAATTGAAAGATCTTCAGTGCCTAGAAGATGAACTTGGACCTCTGCGGCATGTTCTGGATTTGACTCAAAGCAAAAGCTTTCAATTGGAAGATGCTGAGAATTTCATCAGCAATATCAGAGTAACTGTTGTAAAACTAAAGGGCTCTGACAACACATTTGAGTGCCAATTCGATGATGAGTCAGCAACTGTGGTGGACTTTCTGAGGAGATGGATAGCCTTCTGTCAAAGCATCATCTCAACAAGCCCTCAATAA

5. plasmid vehicle: pUC57 backbone plasmid

The plasmid was only used for mouse-derived related experiments. Other species need to replace the sequence of related proteins.
